# Condition-dependent migratory behaviour of endangered Atlantic salmon smolts moving through an inland sea

**DOI:** 10.1093/conphys/cow018

**Published:** 2016-05-23

**Authors:** Glenn T Crossin, Bruce G Hatcher, Shelley Denny, Kim Whoriskey, Michael Orr, Alicia Penney, Frederick G Whoriskey

**Affiliations:** af1 Department of Biology, Dalhousie University, Halifax, Nova Scotia, Canada; af2 Bras d’Or Institute for Ecosystem Research, Cape Breton University, Sydney, Nova Scotia, Canada; af3 Unima’ki Institute of Natural Resources, Eskasoni, Nova Scotia, Canada; af4 Department of Mathematics and Statistics, Dalhousie University, Halifax, Nova Scotia, Canada; af5 Ocean Tracking Network, Dalhousie University, Halifax, Nova Scotia, Canada

**Keywords:** Acoustic telemetry, body condition, juvenile, migration, ocean tracking network, *Salmo salar*, temperature

## Abstract

The Bras d’Or Lake watershed of Cape Breton Island, Nova Scotia, Canada is a unique inland sea ecosystem, UNESCO Biosphere Reserve and home to a group of regionally distinct Atlantic salmon (*Salmo salar*) populations. Recent population decreases in this region have raised concern about their long-term persistence. We used acoustic telemetry to track the migrations of juvenile salmon (smolts) from the Middle River into the Bras d’Or Lake and, subsequently, into the Atlantic Ocean. Roughly half of the tagged smolts transited the Bras d’Or Lakes to the Atlantic Ocean, using a migration route that took them through the Gulf of St Lawrence’s northern exit at the Strait of Belle Isle (∼650 km from the home river) towards feeding areas in the Labrador Sea and Greenland. However, a significant fraction spent >70 days in the Lakes, suggesting that this population has an alternative resident form, in which smolts limit their migrations within the Bras d’Or. Smolts in good relative condition (as determined from length-to-mass relationships) tended to be residents, whereas fish in poorer condition were ocean migrants. We also found a covarying effect of river temperature that helped to predict residence vs. ocean migration. We discuss these results relative to their bioenergetic implications and provide suggestions for future studies aimed at the conservation of declining salmon populations in Canada.

## Teaser

We used acoustic telemetry and biological sampling of Atlantic salmon smolts to reveal the migration tactics that fish use when migrating through a unique brackish sea ecosystem in Canada. We found an interacting effect of water temperature and fish condition that predicted whether fish migrated to sea.

## Introduction

North American Atlantic salmon (*Salmo salar*) populations have been decreasing throughout the species’ range since European colonization (Whoriskey, 2009). However, acute decreases at the southern margins of their distribution (Gibson *et al.*, 2006) are pushing many populations close to extinction (Committee on the Status of Endangered Wildlife in Canada, 2011). It is clear that a reduction in sea survival is limiting salmon populations (Dutil and Coutu, 1988; Holm *et al.*, 2000; Lacroix and Knox, 2005; Lacroix *et al.*, 2005; Halfyard *et al.*, 2012), but the mechanisms underlying reduced survival are uncertain. The synchronous and geographically widespread decline of many different populations might suggest a common driver (Mills *et al.*, 2013; see also Springer and van Vliet, 2014 for Pacific salmon). However, given the pronounced phenotypic diversity among Atlantic salmon populations, which influences many aspects of their ecology (Ritter, 1989; O’Connell *et al.*, 2006), no satisfactory explanation for the observed and wide-spread decreases has yet emerged (Halfyard *et al.*, 2012, 2013; Friedland *et al.*, 2014). Researchers are now focusing on understanding the determinants of marine survival in regional population groupings that differ markedly in their life history and migration tendencies (e.g. Lacroix *et al.*, 2005; O’Connell *et al.*, 2006; Kocik *et al.*, 2009; Halfyard *et al.*, 2013). It is hoped that with enough of this kind of information on behaviour and survival, we will eventually be able to identify either a common driver that affects all salmon population trends or various drivers acting regionally or at population-specific scales.

The early, freshwater life-history stage and the transition between the freshwater and marine life-history phases (smolting) are the first significant survival challenges for juvenile salmon (e.g. the ‘mortality bottleneck’; Kennedy *et al.*, 2008; Crossin *et al.*, 2015), but these are difficult to measure. Individual survival and its population-level consequences can now be studied with relative ease via electronic tracking techniques (Hussey *et al.*, 2015). From the limited number of telemetry studies of Atlantic salmon smolts, as well as smolts of other anadromous salmonids, it is apparent that survival estimates during the transition to marine environments varies with population and species. Predation appears to be an important factor in many but not all populations, whereas local environmental conditions that either enhance or reduce predation risk may be the most crucial determinant of survival (Lacroix *et al.*, 2005; Kocik *et al.*, 2009; Halfyard *et al.*, 2013).

Salmon smolts originating from Cape Breton in eastern Canada migrate through the Bras d’Or Lake ecosystem *en route* to the Atlantic Ocean. However, the salmon in this ecosystem may have a fundamentally different biology from Atlantic salmon elsewhere. The Bras d’Or Lake is the only brackish-water, inland sea in North America, a distinction that warranted designation as a World Biosphere Reserve by United Nations Educational, Scientific and Cultural Organization (UNESCO) in 2011. Drawing from thousands of years of aboriginal traditional ecological knowledge, members of the Mi’kmaq First Nation hypothesize that at least a portion of the salmon originating from the rivers of the Bras d’Or close their life history within the Pitu’pak (the Mi’kmaq name of the Bras d’Or system; Collaborative Environmental Planning Initiative, 2006). This would mean that some salmon do not migrate to the Northwest Atlantic as most other salmon do elsewhere, despite having direct access to the ocean. This raises intriguing questions about the evolution of salmon in this system, and whether Bras d’Or salmon possess alternative migratory phenotypes (*sensu*Hutchings, 1986). Generally, salmon smolts use estuaries to a minimal extent during seaward migration, transiting in a directed, expeditious manner (Moore *et al*., 1998; Lacroix *et al.*, 2005; Thorstad *et al.*, 2011; Lefèvre *et al.*, 2012). However, the hypothesis that salmon close their life history within the Bras d’Or ecosystem has not been formally tested, and so the importance of this habitat is not clear.

The timing of smolt migrations down natal rivers and into estuaries is likely to be cued by both endogenous and exogenous factors. Body condition (body mass, somatic lipid density, etc.) is an important factor for the initiation of smoltification (Rikardsen *et al.*, 2004). Once smoltification is initiated, body condition tends to decrease as somatic energy is directed towards the restructuring of osmoregulatory systems, continued growth and locomotion (Wedemeyer *et al.*, 1980). Body condition may therefore influence individual migration tendency, as well as rates of travel and the timing of entry into the marine environment.

Water temperature is also a robust predictor of the timing of smolt migration and can operate either independently from, or in tandem with, other environmental factors, such as river discharge rates, water velocity, turbidity and lunar cycles (McCormick *et al.*, 1998; Thorstad *et al.*, 2011). As an abiotic ‘master factor’ that influences many aspects of physiology, behaviour and ecology (Brett, 1971), water temperature alone explains a high proportion of the annual variance of the timing of smolt migration in some rivers (e.g. >90%; Jonsson and Ruud-Hansen, 1985). An interaction between environmental condition and body condition should therefore predict both the timing and the pace of smolt migrations, within a specific seasonal window of opportunity (e.g. the ‘smolt window’; McCormick *et al.*, 1998).

Drawing both from traditional Mi’kmaq knowledge and from our understanding of the ecological and physiological drivers of smolt migrations, our aim was to use acoustic telemetry to document the migratory strategies of fish from this region, by identifying the proportions of down-river migrating smolts that remain within the Bras d’Or estuary vs. migrating to the North Atlantic Ocean. By so doing, we provide initial information about whether salmon possess alternative migratory phenotypes in this unique ecosystem, at least at the juvenile life-history stage. We document spatio-temporal patterns of residency within the Bras d’Or estuary, general migration pathways through the Lakes, the role of body condition on these patterns and the interacting role of temperature. For smolts that migrated to the North Atlantic, we also assess rates of marine travel and overall survival.

## Materials and methods

### Study site, capture methods and fish handling

**Figure 1:**
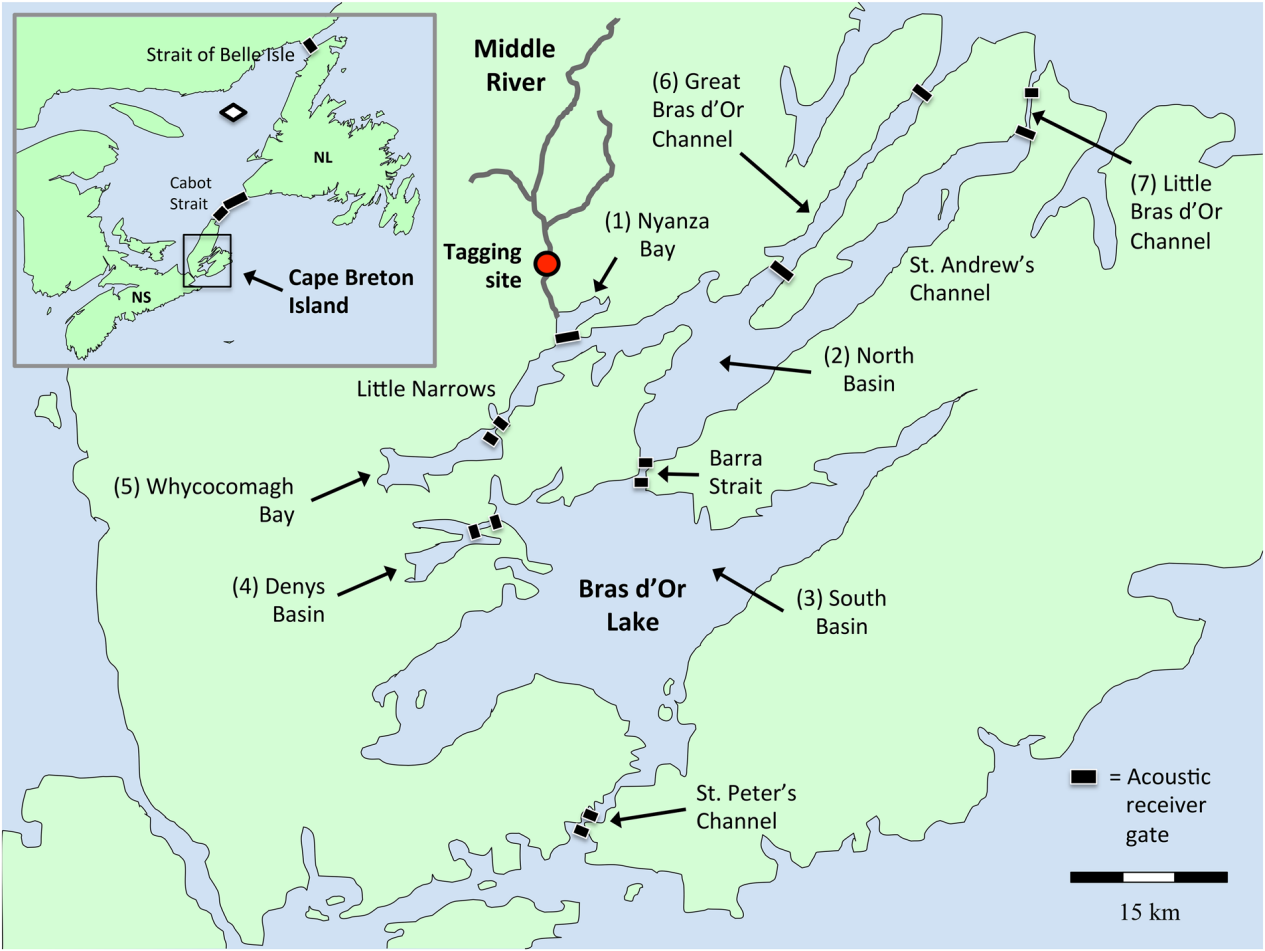
Map of the Ocean Tracking Network’s Bras d’Or Array in Cape Breton Island, Nova Scotia, with an inset of Atlantic Canada. Positions of the various acoustic receiver gates define the boundaries of each numbered segment of the estuary. The white diamond in the inset shows the location of the autonomous Wave Glider in the Gulf of St Lawrence.

The Bras d’Or ‘Lake’ is a hydrodynamically complex inland sea and a UNESCO Biosphere Reserve that dominates the landscape of Cape Breton Island, Nova Scotia (45°51′37″ N, 60°46′44″ W; Fig. 1). It has a surface area of ∼1200 km^2^, drains a watershed area of ∼3600 km^2^, reaches a maximal depth of 280 m and has three narrow channels connecting it to the Atlantic Ocean (Yang *et al.*, 2007). The Middle River (Fig. 1) is the largest river draining into the Bras d’Or, with its main stem running ∼35 km from the Cape Breton Highlands and draining an area of 325 km^2^. Historically, the Middle River produced the most salmon in this system, with thousands of spawning adults per year (Gibson *et al.*, 2006). More recently (1983–2010), censuses have documented only 31–486 adults, prompting an endangered classification (Gibson *et al.*, 2006).

**Figure 2:**
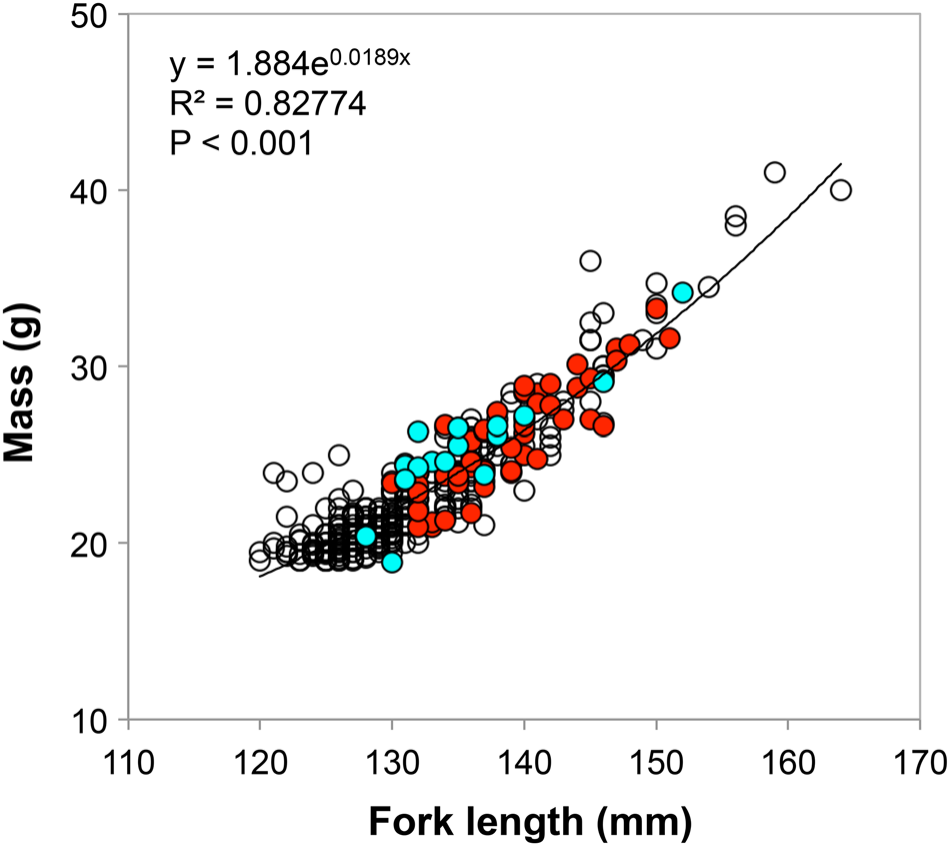
Length–mass relationship for the Atlantic salmon smolts captured on the Middle River, Nova Scotia. Open circles represent all non-tagged smolts captured in both years of study. Red circles indicate the fish tagged in 2013, whereas blue circles are for 2012.

For this study, smolts were captured in the Middle River in partnership with the Unima’ki Institute of Natural Resources (UINR). Smolts were captured with a rotary trap and tagged with acoustic transmitters. In 2012, 16 smolts were captured between 16 and 23 May and implanted with V9 acoustic transmitter tags (2.9 g in air; Vemco Ltd, Halifax, Nova Scotia, Canada). In 2013, 50 smolts were captured between 7 and 11 May and implanted with smaller V8 tags (2.0 g in air). Tag mass was ∼11% of fish body mass in 2012 and ∼8% in 2013. For each fish, we measured fork length (in millimetres) and body mass (±0.1 g), and a few scales were collected for ageing. Fork lengths and body masses were also determined for the smolts that were not selected for tagging (*n* = 150 in 2012 and *n* = 515 in 2013), to provide a comparison to the tagged group (Fig. 2). We used standard surgical procedures to insert the tags in the abdominal cavity (Lacroix *et al.*, 2005). Only fish >128 mm were tagged because their larger sizes more easily accommodated acoustic tags. In both years, all tags were programmed to ping at ∼50 s intervals, which provided a battery life of ∼74 days.

All of our fish handling and surgical protocols conformed to guidelines established by the Canadian Committee on Animal Care, approved by the Dalhousie University Animal Care Committee (Dalhousie University Animal Care Protocol 14–105) and Cape Breton University Animal Care Committee (Permits # ACC-2011-10; ACC#1213-16).

### Acoustic receivers

A spatial array of omni-directional, data-logging acoustic receivers (VR2W; Vemco Ltd) was configured to record patterns of movement, residence and habitat use within the Bras d’Or estuary (Fig. 1) and to detect the directional exit of smolts to the Atlantic Ocean. A total of 25 receivers was deployed within and across the various constrictions that link the main basins and embayments of the Bras d’Or system, as well as the three exits to the Atlantic Ocean. The receivers were placed so that their reception ranges covered the width of the channels or embayments where they were placed (Fig. 1; see Supplementary material for range-testing information). These receiver ‘gates’ were placed near each end of a channel, so that fish moving in a particular direction would be heard first entering and then leaving the channel.

We divided the estuary into seven discrete segments based on their geomorphology, hydrography and water residence times (numbered 1–7 in Fig. 1), and classified a fish as being present in a particular habitat segment when it was detected on receiver gates that bounded that segment. When a fish released at the tagging site on the Middle River was subsequently detected on the Nyanza Bay receiver line, then we classified the fish as having occupied the Nyanza Bay segment of the Bras d’Or. However, if a fish released from the tagging site went undetected on the Nyanza Bay line (or on any other receiver line), we then assumed that the fish either died (possibly predation) somewhere between the release site and the mouth of Nyanza Bay or that it remained resident in Nyanza Bay but out of detection range (although death would be the most likely explanation given the 74 day nominal tag life). Next, if a fish was detected on the Nyanza Bay line and then subsequently on the inner Great Bras d’Or Channel line, the inner Little Bras d’Or Channel line or northern Barra Strait line, we classified the fish as having occupied the North Basin. If, however, the fish was detected on the Nyanza Bay line but then nowhere else, we assumed that it remained resident in Nyanza Bay (at least for the duration of its transmitter’s battery life), although it might have resided or died in other parts of the St Andrew’s Channel of North Basin without being detected. Finally, if a fish was detected on one of the terminal receiver lines positioned at the exits of the Bras d’Or system to the Atlantic Ocean (e.g. Great Bras d’Or Channel, Little Bras d’Or Channel and St Peter’s Channel), then we assumed that it left the Bras d’Or and entered the ocean. We made all of these assumptions based on previous studies, which have shown that Atlantic salmon smolts typically pass rapidly though estuaries, proceeding directly towards the ocean, with infrequent reversals of direction (Thorstad *et al.*, 2011; Halfyard *et al.*, 2013).

Additional detections of tagged fish from the Gulf of St Lawrence occurred on Ocean Tracking Network (www.oceantrackingnetwork.org) acoustic receivers deployed in arrays (i.e. Cabot Strait, Strait of Belle Isle) or mounted on a marine autonomous vehicle [Liquid Robotics (Sunnyvale, CA, USA) Wave Glider SV2] in the Gulf of St Lawrence (Fig. 1).

### Gate detection efficiency

Conclusions about the movement of fish among various segments of the estuary assume that fish will always be detected when they pass a gate. We assessed detection efficiency as follows: (i) by calculating the probability that an individual receiver would detect a single transmission from a fixed or mobile test tag; (ii) by computing the probability that a gate will detect one or more transmissions from a tagged salmon smolt that is known to have passed within range of a gate because of independent detections by another fixed or mobile receiver; (iii) by deploying sentinel tags that signalled at known intervals over extended periods within range of gates located in problematic areas, so we could determine the fraction of signals recorded; or (iv) by conducting range testing, in which tags similar in signalling power to those used on the smolts were periodically deployed from a boat at known distances from receivers to provide measures of detection efficiency at the time of the test (Kessel *et al.*, 2014).

### Data analyses

In the absence of direct measurement of somatic fat or somatic energy density, the residuals from regressions of body mass against a linear measure of body size is the most common method to estimate the body energy condition of fishes (Schulte-Hostedde *et al.*, 2005). We regressed body mass against body length and used the residuals as a measure of length-corrected condition.

Using the acoustic telemetry detection data from the tagged fish, we estimated residence times within each segment of the Bras d’Or Lakes (as numbered in Fig. 1). A two-factor analysis of covariance (ANCOVA) was used to examine segment differences in residency times between fish that exited the Bras d’Or vs. those that did not, in both years, while accounting for variation in tag release date (Julian day). Tukey’s honest significant difference test was used for pairwise comparisons. Likewise, variation in body size (length and mass) was examined. We compared differences in both length and mass as a function of Bras d’Or residency or exit and year of sampling.

To explore relationships between river temperature and salmon abundances (measured as numbers of smolts captured at the fish wheel), we used linear regression. To test the combined influence of water temperature (exogenous factor) and body mass (endogenous factor) as predictors of resident vs. ocean migration strategies, we used a generalized liner model (GLM) with a binomial distribution and logit fit.

Before calculating marine migration rates [body lengths (BL) per second], we applied a growth correction factor of 1.65 mm day^−1^ (Dutil and Coutu, 1988) to the measured smolt lengths at the time of tagging to account for growth occurring between the time of initial fish capture and exit from the Bras d’Or Lakes.

## Results

### Tag detection efficiencies in the Bras d’Or Array

Minimum detection efficiencies at the acoustic gates varied between 82.4 and 96.6% during the study, indicating a high capability of the receiver network to detect our tagged fish. The lowest detection efficiencies occurred for low-power range-testing tags near the surface in shallow, narrow, winding channels when currents were strong and environmental conditions were likely to generate high levels of ambient noise. At least three tagged smolts passed through two of the gates without being detected during the course of the experiments; one through the northern Barra Strait, and two through the outer Little Bras d’Or Channel (during a period when it was briefly compromised in 2013; see Supplementary Data).

Overall, the acoustic receiver array in the Bras d’Or estuary exhibited a high degree of efficiency, a performance that was comparable or superior to that achieved in arrays deployed in environments of similar size and complexity (Kessel *et al.*, 2014).

### Biological characteristics of salmon smolts

An analysis of covariance model comparing length-dependent body mass between years was significant (ANCOVA, *F*_2,64 _= 75.55, *P* < 0.001), but the categorical year effect was not (*F* = 1.98, *P* = 0.164). This means that neither body mass nor the allometric relationship between length and mass differed between years, and that the significance of the full model was driven simply by the covarying effect of body length on body mass (*F* = 148.62, *P* < 0.001). The mean sizes of acoustically tagged fish in both years (mean ± SD: 2012, 135.8 ± 6.2 mm and 25.4 ± 4.2 g; 2013, 138.8 ± 5.3 mm and 26.0 ± 5.6 g) were larger than those of the general population (2012, 130.6 ± 7.3 mm and 22.6 ± 3.4 g; 2013, 131.4 ± 7.6 mm and 20.7 ± 4.1 g; ANCOVA, *F*_3,729_ = 2121.6, *P* < 0.001; Fig. 2).

### Bras d’Or residency and proportions migrating to the Atlantic Ocean

Acoustic tag detection data for both 2012 and 2013 are summarized in Table 1. Four of 16 (25%) smolts tagged in 2012 migrated into the Atlantic Ocean (termed ocean migrants) during the study period, as did 22 of 50 (44%) of those tagged in 2013. Conversely, four of 16 (25%) remained active within the Bras d’Or receiver array throughout the duration of battery life (termed residents) in 2012, as did 18 of 50 (26%) in 2013. In each year, there were fish that were undetected and thus classified as dead, presumably via predation: eight of 16 (50%) in 2012, and 10 of 50 (20%) in 2013.

**Table 1:** Summary of Atlantic salmon (*Salmo salar*) smolt tagging effort and tracking results

Year	No. of tagged smolts	Undetected; presumed mortalities	Detected within array	Resident in Bras d’Or Lake estuary	Ocean migrants	Detected at sea^a^
2012	16	8 (50%)	8 (50%)	4	4	2
2013	50	10 (20%)	40 (80%)	16	24	16

Values are numbers of tagged individuals, and values in parentheses are the percentage of all tagged individuals in a given year. ^a^Marine detections occurred at Cabot Strait and at the Strait of Belle Isle, as well as on the autonomous Ocean Tracking Network Wave Glider. See Fig. 1 for geographical details.

**Figure 3:**
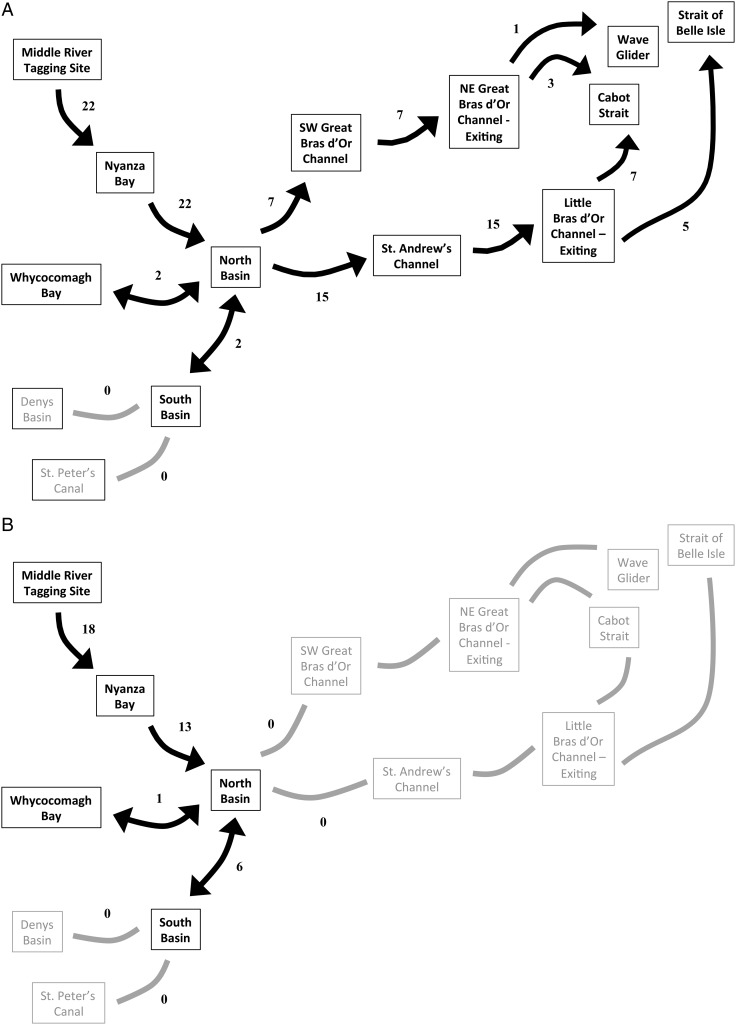
Summary of the acoustic detection data from Atlantic salmon smolts tagged in the Bras d’Or Array during 2013. (**A**) Movements of the 22 tagged smolts that migrated to the Atlantic Ocean. (**B**) Movements of the 18 fish that did not exit the estuary. Grey symbols represent possible migration pathways that were not used by tagged fish.

Patterns of movement within the Bras d’Or were similar between years (although for ease of interpretation, data from 2013 only are presented in Fig. 3). In both years, ocean migrants and residents consistently differed in their occupancy patterns of the different parts of the Bras d’Or system. In 2013, all ocean migrating fish were registered in Nyanza Bay (*n* = 22 of 22) and then subsequently at other locations in the Bras d’Or Array (Fig. 3A). In contrast, only 13 of the 18 resident fish registered in Nyanza Bay (Fig. 3B) were then detected anywhere else in the Bras d’Or. These 13 either remained in parts of the Nyanza Bay or North Basin but out of detection range, or they potentially died via predation or some other cause.

**Figure 4:**
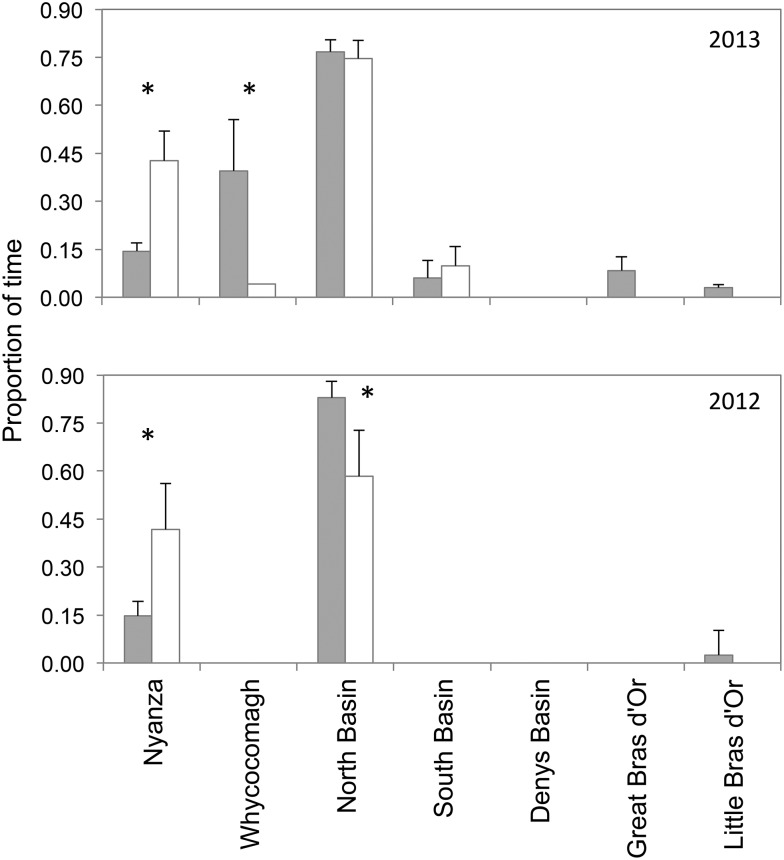
Comparison of Atlantic salmon smolt residence times in each geographical segment of the Bras d’Or Lake estuary in both years of study. Bars indicate least squares means + SEM. Grey bars represent ocean migrants and open bars resident fish (i.e. smolts that stayed in the estuary for the tag battery duration at least). Asterisks indicate statistically significant contrasts between migrants and residents (α = 0.05).

Overall residency times for both the resident and ocean migrant groups were similar in the Bras d’Or, but the two groups differed in the habitats that they occupied (Fig. 4). In both years, resident fish spent significantly more time in Nyanza Bay than ocean migrants (two-factor ANOVA, *F*_2,47_ = 5.916, *P* = 0.005), whereas in 2013 ocean migrants spent significantly more time in St Patrick’s Channel and Whycocomagh Bay than did resident fish (*P* = 0.042), although only three fish (two migrants and one resident) used Whycocomagh Bay in 2013. Finally, in both years, resident and sea migrant fish spent similar amounts of time in the North Basin (*P* = 0.124).

Overall, ocean migrating smolts spent a mean of 30.7 days in the Bras d’Or system before entering the North Atlantic Ocean. Ocean entry coincided with the New Moon, with last detections generally occurring before dusk on an ebbing tide.

### River temperature trends

Water temperatures measured at the fish wheel were similar during the smolt run in both years. They averaged 8.8 ± 1.6°C (mean ± SD) in 2012 and 8.3 ± 1.7°C in 2013. Smolt abundances peaked at the wheel at similar dates in both years (e.g. 10–12 May). The number of fish captured on a given day was positively related to daily river temperature (2012, *F*_1,16_ = 40.29, *P* < 0.001; 2013, *F*_1,20_ = 18.72, *P* = 0.003).

### Endogenous and exogenous correlates of migration behaviour and residency times

**Figure 5:**
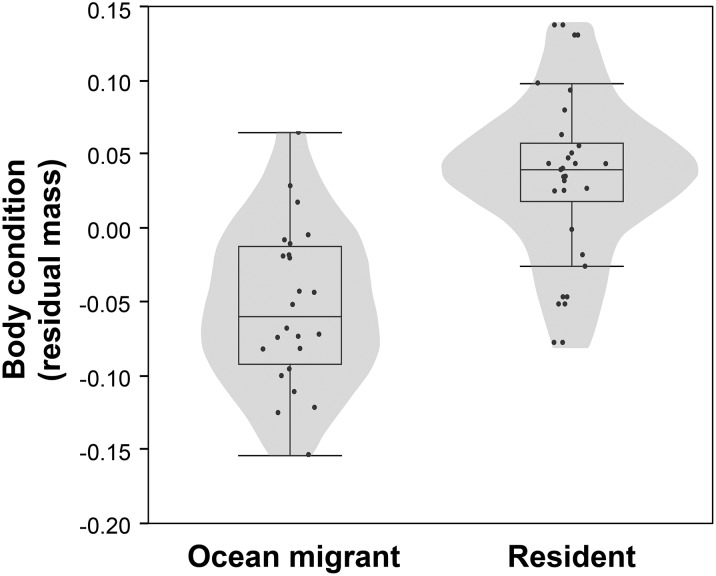
Violin and box plots comparing the body condition residuals (see *Materials and methods*) of Atlantic salmon smolts that either migrated to sea or remained resident within the Bras d’Or Lakes. There were no differences between years; therefore, data were pooled.

**Figure 6:**
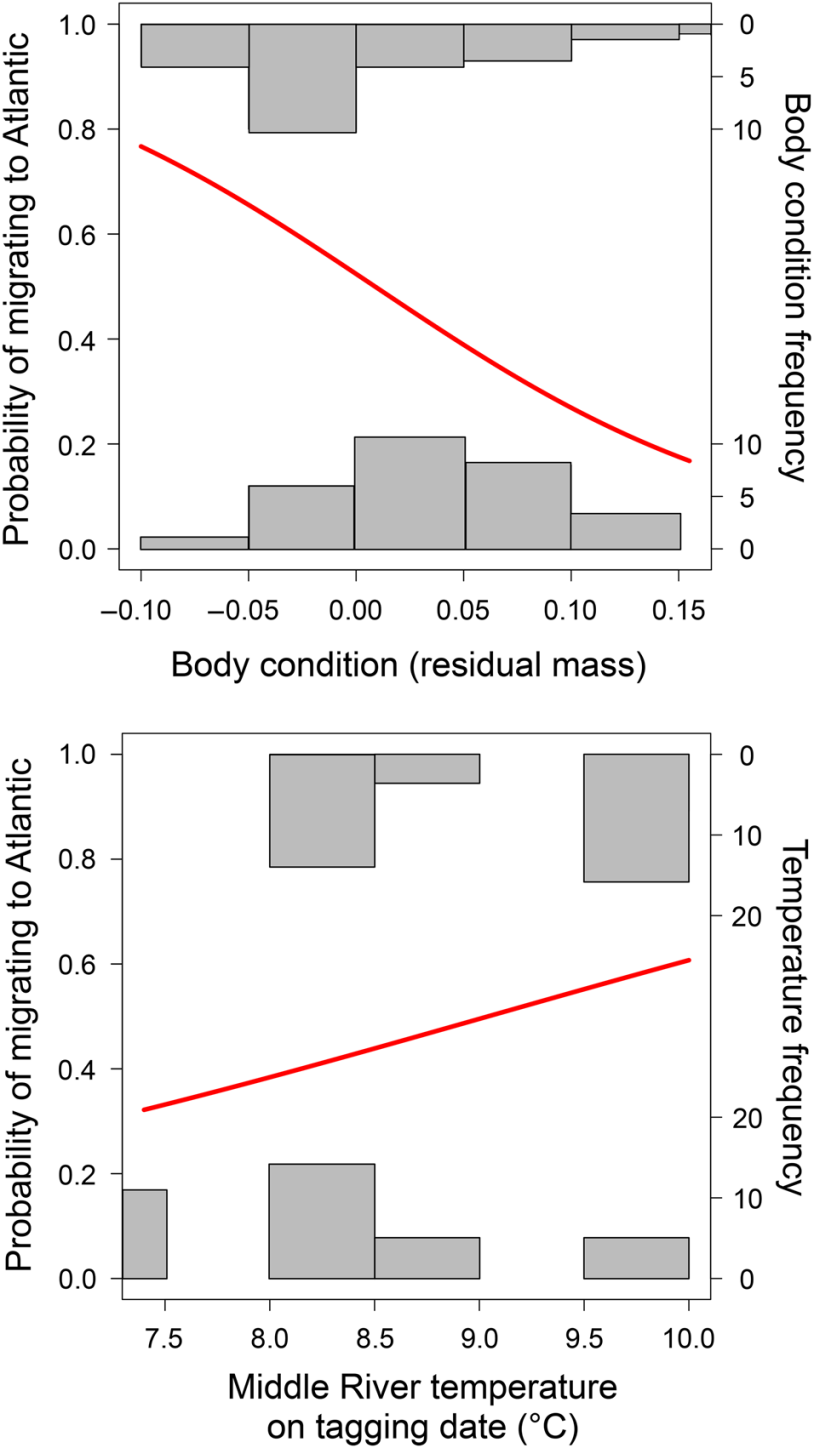
Predictive effect of Atlantic salmon smolt body condition (negative) and river temperature (positive) on the likelihood of migration to the Atlantic Ocean. See Table 2 for statistical output of GLM.

Irrespective of year, body condition differed significantly between resident and ocean migrant smolts [two-factor ANCOVA; *F*_3,62_ = 39.28, *P* < 0.001; resident, 26.1 ± 0.01 g (mean ± SEM), ocean migrant, 25.2 ± 0.01 g; Fig. 5]. Collectively, body condition and river temperature at the time of tagging significantly predicted the likelihood that smolts would migrate to sea or remain resident within the Bras d’Or estuary. The probability of migrating to sea increased when individual fish had low body condition and when river temperatures were warmer (Table 2 and Fig. 6).

**Table 2:** Nominal logistic regression model examining the combined role of endogenous and exogenous factors in predicting whether Atlantic salmon (*S. salar*) smolts remained resident in the Bras d’Or Lake ecosystem or whether they exited to the Atlantic Ocean

Model effects^a^	Estimate	LR χ^2^	*P*-value
Year	−0.991	3.49	0.617
Body condition (residual mass)	−**24.570**	**24.00**	**<0.001**
River temperature	**1.003**	**4.15**	**0.046**

^a^Significant model effects are indicated by bold text. Abbreviations: LR χ^2^, likelihood ratio χ^2^ value. Note that the interaction term (body condition * river temperature) was non-significant and thus removed from the final model to preserve degrees of freedom and increase statistical power. Model output is plotted in Fig. 6.

**Figure 7:**
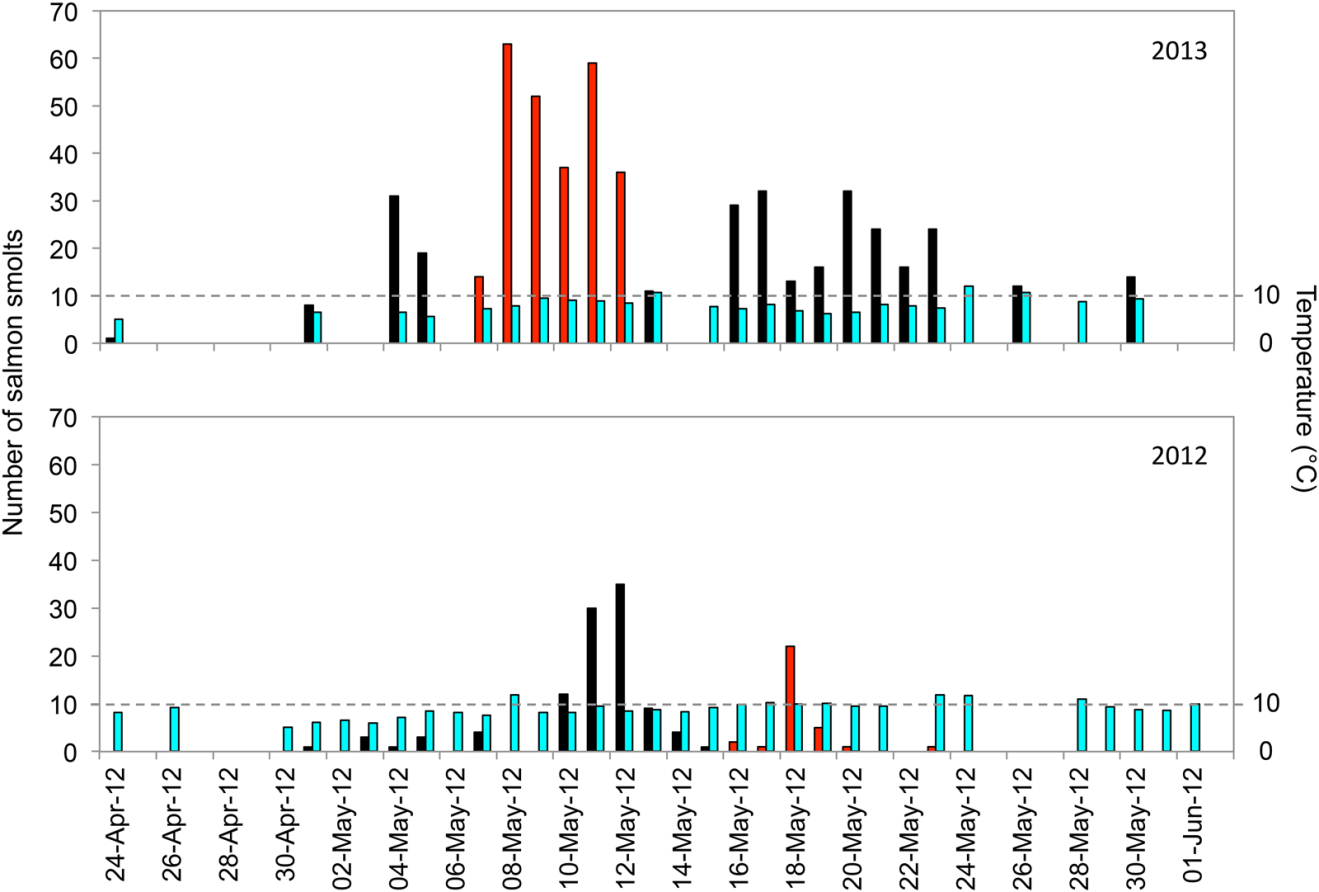
Daily counts of Atlantic salmon smolts captured at the fish wheel on the Middle River, Nova Scotia, and daily river temperatures. Red bars indicate those captured during the period of fish tagging.

River temperature fluctuated during the period of tagging in early May. Overall, temperature rose throughout the season (Fig. 7).

### Marine travel times

Table 3 summarizes the detection and migration rates of smolts on the two marine acoustic lines at Cabot Strait and the Strait of Belle Isle, as well as the lone detection made by the OTN Wave Glider that was sailing transects in the northern Gulf of St Lawrence during the study period. Average travel rates of the fish in our study over the ∼85 km between the Bras d’Or exits and the Cabot Strait line were 50.9 cm s^−1^, equivalent to 2.6 BL s^−1^, which is well within the range of observed migration speeds for post-smolt Atlantic salmon (Folmar and Dickhoff, 1980; Booth *et al.*, 1997; Kocik *et al.*, 2009; Lefèvre *et al.*, 2012).

**Table 3:** Summary of calculated ocean migration speeds for Atlantic salmon (*S. salar*) post-smolts leaving the Bras d’Or Lake estuary of Cape Breton

Year	BdL exit route	Days to exit	Corrected body length (mm)^a^	To CBS (km day^−1^ | BL s^−1^)	To SBI (km day^−1^ | BL s^−1^)	To OTN-WG (km day^−1^ | BL s^−1^)
2012	LBC	33.7	195	49.7 | 3.0	42.8 | 2.6	
	LBC	33.7	194	37.2 | 2.2		
2013	LBC	42.1	216	93.6 | 5.0		
	LBC	38.0	194	59.2 | 3.5		
	LBC	31.0	195	53.9 | 3.2		
	LBC	39.5	206	43.5 | 2.4		
	LBC	31.3	186	36.7 | 2.3		
	LBC	36.2	201	31.3 | 1.8		
	LBC	31.4	184	21.5 | 1.4		
	LBC	26.2	179		22.4 | 1.4	
	LBC	33.2	188		21.8 | 1.3	
	LBC	31.3	190		21.6 | 1.3	
	LBC	27.1	195		21.1 | 1.3	
	LBC	26.2	194		16.4 | 1.0	
	GBC	33.5	201	38.0 | 2.2		
	GBC	30.3	189	35.9 | 2.2		
	GBC	29.5	183	27.8 | 1.8		
	GBC	52.1	218			52.1 | 2.8

Distances used to calculate swimming speeds assumed the shortest straight-line distances between the last detection point in the Bras d’Or Array and the first detection point in the Atlantic Ocean (see Fig. 1). Abbreviations: BdL, Bras d’Or Lake estuary; BL, body length; CBS, Cabot Strait acoustic receiver gate; GBC, Great Bras d’Or Channel; LBC, Little Bras d’Or Channel; OTN-WG, Ocean Tracking Network Wave Glider; SBI, Strait of Belle Isle acoustic receiver gate. ^a^The initial length of smolts when measured at tagging was adjusted to account for the additional growth occurring between the time of tagging and exit from the estuary (days to exit), based on a post-smolt growth rate of 1.65 mm day^−1^ (estimated for Gulf of St Lawrence populations by Dutil and Coutu, 1988).

## Discussion

In this study, acoustic telemetry showed that 25–44% of the Atlantic salmon smolts originating from the largest river in the Bras d’Or watershed successfully made directed marine migrations into the Atlantic Ocean and onwards through the Cabot Strait and the Strait of Belle Isle, towards known foraging areas in the Labrador Sea and western Greenland (Fig. 1; Mills, 2003). However, a similar fraction of the smolts were not detected exiting the Bras d’Or Array, which at least partly supports the hypothesis that some individuals may have adopted a local residency strategy as an alternative to ocean migration, at least during the ∼74 day battery lifespan of the acoustic tags used in this study. However, most North American salmon populations containing two sea-winter individuals (i.e. spending two winters at sea prior to first spawning) are thought to make extensive marine migrations to productive foraging areas near Labrador, Greenland and Iceland (Mills, 2003). In contrast, one sea-winter fish tend to adopt more localized migration strategies, such as those in the Inner Bay of Fundy (Lacroix and Knox, 2005; Lacroix, 2008). However, whether the resident smolts that had delayed ocean migration for >72 days remain resident indefinitely is unknown. To test whether fish remain resident, and thus adopt an estuarine strategy, smolts would need to be tagged with acoustic transmitters programmed to last for at least a year.

The large fraction of tagged smolts that moved rapidly out to sea indicates that the ocean migrant life-history option is at least as important in this population as a resident strategy. Future work will examine the inter-individual variation in migration vs. residency patterns, explore the environmental and endogenous correlates that underlie this variation and examine how the two strategies could potentially affect population persistence and resilience.

### Overall survival, and Bras d’Or residency vs. ocean migration

The emigration of Atlantic salmon smolts from freshwater to estuaries can be a period of naturally high predation mortality, with estimates ranging from 0 to >50% (Hvidsten and Lund, 1988; Dieperink *et al.*, 2002; Thorstad *et al.*, 2011; Lefèvre *et al.*, 2012). If we assume that non-detection of fish in the Bras d’Or Array equates to mortality, then the mortality estimate for 2012 (50%) was higher than in 2013 (20%). The higher mortality in 2012 compared with 2013 could be attributable to a burden imposed by the use of a larger model tag (V9, ∼11% of body mass vs. V8, ∼8% in 2013). However, a growing number of studies report that for some fishes, including Atlantic salmon, there are no significant mortality, tag loss or sublethal impacts of surgical tagging when devices are <12% of body mass (Brown *et al.*, 1999), hence the inter-year differences probably reflect variation in predation between years (e.g. Halfyard *et al.*, 2012, 2013; Jonsson and Jonsson, 2014).

Once past the fresh–salt transition zone at the Middle River delta, we found that there were some distributional differences between resident and ocean-migrating smolts. Resident fish spent proportionally more time in Nyanza Bay than did ocean migrating fish. This suggests that despite their better body condition, residents may have required additional time to become fully salt tolerant (e.g. up-regulated gill Na^+^/K^+^-ATPase activity, number and size of gill chloride cells, and intestinal water permeability; Boeuf, 1993) and may therefore have spent more time within the freshwater zones near river mouths. Conversely, ocean migrating fish may have already been sufficiently hypo-osmoregulated, permitting them to move into the estuary without delay. Once through Nyanza Bay, two of the ocean migrants spent more time in Whycocomagh Bay than did the one estuary resident fish (see Fig. 3), suggesting perhaps that fish in poor condition may find suitable habitat for growth or foraging there.

There was an important covarying effect of body condition and temperature in the decision to remain resident or migrate to sea. In both years, decreased body condition increased the probability that smolts would migrate, whereas better-conditioned smolts remained resident. Coupled with the previous observation that ocean migrants moved from the Middle River and into the Bras d’Or Lake estuary at a faster pace than resident fish, migrants were perhaps more fully prepared for salt water tolerance. As the energetic condition of ocean migrants was also more depleted than residents, owing in part to the unavoidable cost of investing in osmoregulatory restructuring for saltwater entry, this suggests a greater urgency for migrants to get to sea, where foraging opportunities are generally more favourable than in the Bras d’Or, and thus better suited for restoring condition. Similar trends have been observed in Atlantic salmon elsewhere (Halttunen *et al.*, 2012), and in closely related brown trout (Eldøy *et al.*, 2015) and brook charr (Thériault and Dodson, 2003 and references therein). Corroborating this, studies have indicated that the Bras d’Or is indeed nutrient limited, with primary productivity below that found in coastal areas of Nova Scotia (Gibson *et al.*, 2014), and lacking the important prey (e.g. *Calanus finmarchicus*) that are found abundantly in the Gulf of St Lawrence and Scotian Shelf (Shih *et al.*, 1988).

Environmentally, river temperature recorded at the time of capture and tagging was also a significant predictor of ocean migration, with higher temperatures increasing the probability of ocean migration. Bioenergetic theory would predict temperature to influence decisions concerning migration (Handeland *et al.*, 2008). When basal metabolism is elevated because of higher ambient temperatures, smolts in poor relative condition would presumably succumb to energetic exhaustion more quickly than smolts in better condition. Migration to the generally cooler and more productive coastal waters would surely bolster survival probability (Hendry *et al.*, 2004).

### Marine migration rates and survival in the North Western Gulf of St Lawrence

The percentage of ocean migrant fish that survived to enter the Gulf of St Lawrence definitively in the 2 years of the study was high, at 69% (18 of 26). Rates of travel between the Bras d’Or and the Strait of Belle Isle at the northern exit of the Gulf averaged 28.2 cm s^−1^ (1.5 BL s^−1^), which is similar to migration rates observed previously in the Gulf of St Lawrence by tagged smolts from the Rivière St Jean, Quebec (Lefèvre *et al.*, 2012). These rates of travel may be indicative of foraging behaviour (Dutil and Coutu, 1988). Interestingly, only one of 12 fish detected on the Cabot Strait line was subsequently detected departing the Gulf through the Strait of Belle Isle. These fish could have died in the Gulf, changed course by moving eastward to the North Atlantic Ocean, their batteries could have died before detection at the Strait of Bell Isle, or they could have eluded detection by the receivers there. An additional six tagged smolts were missed by the receivers on the Cabot Strait line but had clearly crossed it because they were subsequently detected at the Strait of Belle Isle and by a mobile receiver carried by an autonomous vehicle (Liquid Robotics Wave Glider) that was patrolling the waters of the Gulf during the study. This suggests that a pathway through the Gulf is the normal marine migration route for Middle River smolts. Many of the receivers of the Cabot Strait line are positioned close to the bottom in deep water, which decreases their detection range for pelagic fish. Taken together, it seems most likely that survival of the tagged smolts once they entered the Gulf was high, and our low redetection at the Strait of Belle Isle of fish that had previously crossed the Cabot Strait was attributable to expiration of tag batteries and environmental conditions that limited receiver ranges.

### Conclusion

This study has shown definitively that the Atlantic salmon population in the Bras d’Or Lakes does not exclusively close its life history within the Lakes. Instead, nearly half of the salmon tracked rapidly entered the Atlantic Ocean after leaving the Middle River and were detected moving towards known foraging areas near Greenland. Residency times of ocean migrant and resident fish differed to some extent in the Lakes, but future studies are needed to understand why this occurs and the associated patterns of activity and utilization. Nevertheless, the resident vs. ocean migrant strategies that we observed suggest that the brackish inland sea can be an important rearing or staging area for young salmon, which differentiates these Bras d’Or salmon populations from those in other parts of Nova Scotia and eastern Canada. This suggests that the Bras d’Or Lakes are a crucial habitat for juvenile salmon, rather than a habitat that is simply traversed *en route* to feeding areas in the open ocean.

With respect to management of these Atlantic salmon, our results suggest that efforts should be made to incorporate our spatial results into future conservation planning. This would apply to fisheries activities, which should be planned to avoid unintended by-catch in the regions where smolts are found to reside (Fig. 3), and aquaculture currently underway in the Bras d’Or should be planned and monitored so that impacts are minimal. More extensive information about distributions and temporal residence patterns of salmon are still needed, however, so that any regulations do not unnecessarily impinge on the development of the local economy.

## Supplementary material

Supplementary material is available at *Conservation Physiology* online.

## Funding

This work was supported by the Ocean Tracking Network via funding from the Canada Foundation for Innovation, and the Natural Sciences and Engineering Research Council of Canada. Additional support was provided by a Natural Sciences and Engineering Research Council of Canada Discovery Grant to G.T.C.

## Supplementary Material

Supplementary Data

## References

[COW018C1] Boeuf G (1993) Salmonid smolting: a pre-adaptation to the oceanic environment. In Rankin JC, Jensen FB, eds, Fish Ecophysiology. Chapman and Hall, London, pp 105–135.

[COW018C2] Booth RK, Bombardier EB, McKinley RS, Scruton DA, Goosney RF (1997) Swimming performance of post spawning adult (kelts) and juvenile (smolts) Atlantic salmon, *Salmo salar*. Can Manu Rep Fish Aquat Sci 2406: v + 18.

[COW018C3] Brett JR (1971) Energetic responses of salmon to temperature. A study of some thermal relations in the physiology and freshwater ecology of sockeye salmon (*Oncorhynchus nerka*). Am Zool 11: 99–113.

[COW018C4] Brown RS, Cooke SJ, Anderson WG, McKinley RS (1999) Evidence to challenge the 2% rule for biotelemetry. North Am J Fish Manag 19: 867–871.

[COW018C5] Collaborative Environmental Planning Initiative (2006) Bras d’Or Lakes Traditional Ecological Knowledge Workshop Proceedings. Unama’ki Institute for Natural Resources, Eskasoni, NS.

[COW018C6] Committee on the Status of Endangered Wildlife in Canada (2011) Assessment and Status Report on the Atlantic salmon *Salmo salar* in Canada. Ottawa, ON http://www.sararegistry.gc.ca/virtual_sara/files/cosewic/sr_atlantic_salmon_2011_eng.pdf.

[COW018C7] Crossin GT, Sundström LF, Vandersteen WE, Devlin RH (2015) Early life-history consequences of growth hormone transgenesis in rainbow trout reared in stream ecosystem mesocosms. PLoS ONE 10: e0120173. 25807001 10.1371/journal.pone.0120173 PMC4373795

[COW018C8] Dieperink C, Bak BD, Pedersen LF, Pedersen MI, Pedersen S (2002) Predation on Atlantic salmon and sea trout during their first days as postsmolts. J Fish Biol 61: 848–852.

[COW018C9] Dutil JD, Coutu JM (1988) Early marine life of Atlantic salmon, *Salmo salar*, postsmolts in the northern Gulf of St. Lawrence. Fish Bull 86: 197–212.

[COW018C10] Eldøy SH, Davidsen JG, Thorstad EB, Whoriskey F, Aarestrup K, Næsje TF, Rønning L, Sjursen AD, Rikardsen AH, Arnekleiv JV (2015) Marine migration and habitat use of anadromous brown trout (*Salmo trutta*). Can J Fish Aquat Sci 72: 1366–1378.

[COW018C11] Folmar LC, Dickhoff WW (1980) The parr–smolt transformation (smoltification) and seawater adaptation in salmonids: a review of selected literature. Aquaculture 21: 1–37.

[COW018C12] Friedland KD, Shank BV, Todd CD, McGinnity P, Nye JA (2014) Differential response of continental stock complexes of Atlantic salmon (*Salmo salar*) to the Atlantic Multidecadal Oscillation. J Mar Syst 133: 77–87.

[COW018C13] Gibson AJF, Hubley B, Chaput G, Dempson JB, Caron F, Amiro P (2006) Summary of status and abundance trends for eastern Canadian Atlantic salmon (*Salmo salar*) populations. DFO Can Sci Advis Sec Res 2006/026: iv + 31. http//www.dfo-mpo.gc.ca/Library/327918.pdf.

[COW018C14] Gibson AJF, Horsman TL, Ford JS, Halfyard EA (2014) Recovery potential assessment for eastern Cape Breton Atlantic salmon (*Salmo salar*): habitat requirements and availability, and threats to populations. DFO Can Sci Advis Sec Res 2014/071: vi + 141. http//www.dfo-mpo.gc.ca/Library/360157.pdf.

[COW018C15] Halfyard EA, Ruzzante DE, Stokesbury MJW, Gibson AJF, Whoriskey FG (2012) Estuarine migratory behaviour and survival of Atlantic salmon smolts from the Southern Upland, Nova Scotia, Canada. J Fish Biol 81: 1626–1645. 23020565 10.1111/j.1095-8649.2012.03419.x

[COW018C16] Halfyard EA, Gibson AJF, Stokesbury MJW, Ruzzante DE, Whoriskey FG (2013) Correlates of estuarine survival of Atlantic salmon postsmolts from the Southern Upland, Nova Scotia, Canada. Can J Fish Aquat Sci 70: 452–460.

[COW018C17] Halttunen E, Jensen JLA, Næsje TF, Davidsen JG, Thorstad EB, Chittenden CM, Hamel S, Primicerio R, Rikardsen AH (2012) State-dependent migratory timing of postspawned Atlantic salmon (*Salmo salar*). Can J Fish Aquat Sci 70: 1063–1071.

[COW018C180] Handeland SO, Imsland AK, Stefansson SO (2008) The effect of temperature and fish size on growth, feed intake, food conversion efficiency and stomach evacuation rate of Atlantic salmon post-smolts. Aquaculture 283: 36–42.

[COW018C410] Hendry AP, Bohlin T, Jonsson B, Berg OK (2004) To sea or not to sea? Anadromy versus residency in salmonids. In Henry AP, Stearns SC eds, Evolution Illuminated: Salmon and their Relatives. Oxford University Press, Oxford, pp 92–125.

[COW018C18] Holm M, Holst JC, Hansen LP (2000) Spatial and temporal distribution of post-smolts of Atlantic salmon (*Salmo salar*) in the Norwegian Sea and adjacent areas. ICES J Mar Sci 57: 955–964.

[COW018C19] Hussey NE, Kessel ST, Aarestrup K, Cooke SJ, Cowley PD, Fisk AT, Harcourt RG, Holland KN, Iverson SJ, Kocik JF et al (2015) Aquatic animal telemetry: a panoramic window into the underwater world. Science 348: 6240. 10.1126/science.1255642 26068859

[COW018C20] Hutchings JA (1986) Lakeward migrations by juvenile Atlantic salmon, *Salmo salar*. Can J Fish Aquat Sci 43: 732–741.

[COW018C21] Hvidsten NA, Lund RA (1988) Predation on hatchery-reared and wild smolts of Atlantic salmon, *Salmo salar*, in the estuary of River Orkla, Norway. J Fish Biol 33: 121–126.

[COW018C22] Jonsson N, Jonsson B (2014) Time and size at seaward migration influence the sea survival of Atlantic salmon (*Salmo salar* L.). J Fish Biol 84: 1457–1473. 24773540 10.1111/jfb.12370

[COW018C23] Jonsson B, Ruud-Hansen J (1985) Water temperature as the primary influence on timing of ocean migrations of Atlantic salmon (*Salmo salar*) smolts. Can J Fish Aquat Sci 42: 593–595.

[COW018C24] Kennedy BP, Nislow KH, Folt CL (2008) Habitat-mediated foraging limitations drive survival bottlenecks for juvenile salmon. Ecology 85: 2529–2541. 10.1890/06-1353.1 18831174

[COW018C25] Kessel ST, Cooke SJ, Heupel MR, Hussey NE, Simpfendorfer CA, Vagle S, Fisk AT (2014) A review of detection range testing in aquatic passive acoustic telemetry studies. Rev Fish Biol Fish 24: 199–218.

[COW018C26] Kocik JF, Hawkes JP, Sheehan TF, Music PA, Beland KF (2009) Assessing estuarine and coastal migration and survival of wild Atlantic salmon smolts from the Narraguagus River, Maine using ultrasonic telemetry. Am Fish Soc Symp 69: 293–310.

[COW018C27] Lacroix GL (2008) Influence of origin on migration and survival of Atlantic salmon (*Salmo salar*) in the Bay of Fundy, Canada. Can J Fish Aquat Sci 65: 2063–2079.

[COW018C28] Lacroix GL, Knox D (2005) Distribution of Atlantic salmon (*Salmo salar*) postsmolts of different origins in the Bay of Fundy and Gulf of Maine and evaluation of factors affecting migration, growth, and survival. Can J Fish Aquat Sci 62: 1363–1376.

[COW018C29] Lacroix GL, Knox D, Stokesbury MJW (2005) Survival and behaviour of post-smolt Atlantic salmon in coastal habitat with extreme tides. J Fish Biol 66: 485–498.

[COW018C30] Lefèvre MA, Stokesbury MJW, Whoriskey FG, Dadswell MJ (2012) Migration of Atlantic salmon smolts and post-smolts in the Rivière Saint-Jean, QC north shore from rivierine to marine ecosystems. Environ Biol Fish 96: 1017–1028.

[COW018C31] McCormick SD, Hansen LP, Quinn TP, Saunders RL (1998) Movement, migration, and smolting of Atlantic salmon (*Salmo salar*). Can J Fish Aquat Sci 55: 77–92.

[COW018C32] Mills D (2003) Salmon at the Edge. Wiley-Blackwell, Oxford.

[COW018C33] Mills KE, Pershing AJ, Sheehan TF, Mountain D (2013) Climate and ecosystem linkages explain widespread declines in North American Atlantic salmon populations. Glob Chang Biol 19: 3046–3061. 23780876 10.1111/gcb.12298

[COW018C330] Moore A, Ives S, Mead TA, Talks L (1998) The migratory behaviour of wild Atlantic salmon (*Salmo salar*) smolts in the River Test and Southampton Water, Southern England. Hydrobiologia 371/372: 295–304.

[COW018C340] O’Connell MF, Dempson JB, Chaput G (2006) Aspects of the life history, biology and population dynamics of Atlantic salmon in eastern Canada. Can Sci Advis Sec Doc 2006/014: iv + 54. http://www.dfo-mpo.gc.ca/Library/323204.pdf.

[COW018C35] Rikardsen AH, Haugland M, Bjorn PA, Finstad B, Knudsen R, Dempson JB, Holst JC, Hvidsten NA, Holm M (2004) Geographical differences in marine feeding of Atlantic salmon post-smolts in Norwegian fjords. J Fish Biol 64: 1655–1679.

[COW018C36] Ritter JA (1989) Marine migration and natural mortality of North American Atlantic salmon (*Salmo salar*). Can Manu Rep Fish Aquat Sci 2041: x + 136.

[COW018C37] Schulte-Hostedde AI, Zinner B, Millar JS, Hickling GJ (2005) Restitution of mass–size residuals: validating body condition indices. Ecology 86: 155–163.

[COW018C38] Shih C, Marhue L, Barrett N, Munro R (1988) Planktonic copepods of the Bras d’Or Lakes system, Nova Scotia, Canada. Hydrobiologia 167/168: 3319–3324.

[COW018C39] Springer AM, van Vliet G (2014) Climate change, pink salmon, and the nexus between bottom-up and top-down forcing in the subarctic Pacific Ocean and Bering Sea. Proc Nat Acad Sci *USA* 111: E1880–E1888. 24706809 10.1073/pnas.1319089111 PMC4020041

[COW018C40] Thériault V, Dodson JJ (2003) Body size and the adoption of a migratory tactic in brook charr. J Fish Biol 63: 1144–1159.

[COW018C41] Thorstad EB, Whoriskey FG, Rikardsen AH, Aarestrup K (2011) Aquatic nomads: the life and migrations of the Atlantic salmon. In Aas Ø, Einum S, Klemetsen A, Skurdal J, eds, Atlantic Salmon Ecology. Blackwell Publishers, Oxford, pp 1–32.

[COW018C42] Wedemeyer GA, Saunders RL, Clarke WC (1980) Environmental factors affecting smoltification and early marine survival of anadromous salmonids. US Nat Mar Fish Serv Mar Fish Rev 42: 1–14.

[COW018C43] Whoriskey FG (2009) Management angels and demons in the conservation of Atlantic salmon in North America. Am Fish Soc Symp 70: 1083–1102.

[COW018C44] Yang B, Sheng J, Hatcher BG, Petrie B (2007) Numerical study of circulation and temperature-salinity distributions in the Bras d’Or Lakes. Ocean Dynam 57: 245–268.

