# Condition-dependent migratory behaviour of endangered Atlantic salmon smolts moving through an inland sea

**DOI:** 10.1093/conphys/cow032

**Published:** 2016-08-22

**Authors:** Glenn T. Crossin, Bruce G. Hatcher, Shelley Denny, Kim Whoriskey, Michael Orr, Alicia Penney, Frederick G. Whoriskey

doi: 10.1093/conphys/cow018

In the original version of this article, the author affiliation for Shelley Denny was misspelled. It read ‘Unima'ki’, but it should have read ‘Unama'ki’.

The publisher apologises for this error.

