# Supplementary material for: Condition-dependent migratory behaviour of endangered Atlantic salmon smolts moving through an inland sea
Source: Conserv Physiol. 2016 May 23;4(1):cow018. doi: 10.1093/conphys/cow018 (PMC4877435; doi:10.1093/conphys/cow018)
Supplement: Supplementary Data [file cow018supp.zip › cow018supp.docx]

**Conservation Physiology - Manuscript ID CONPHYS-2016-010**

**~ Supplementary Data**

“Condition dependent migration behaviour of endangered Atlantic salmon smolts moving through an inland sea”

*Acoustic Receiver Array Performance*

The acoustic receiver array in the Bras d’Or estuary exhibited a high degree of efficiency overall, which was comparable or superior to that achieved in arrays deployed in environments of similar size and complexity (Kessel et al. 2014). Overall, detection efficiency was greater than 90% occurred in narrow, shallow (<15m water depth) channels that experienced high tidal flows (Table 1). The worst performing gate was that on the outer end of the Little Bras d’Or Channel, where fast, turbulent tidal flows passed through a sharp bend in the channel, and fishing boat traffic is common. This two-receiver gate was compromised by the loss of one receiver sometime between 17^th^ April and 5^th^ June 2013 (the American lobster fishing season spans 15^th^ May to 15^th^ July in Cape Breton). Three tagged fish passed through gates without being detected (Table 1). Two of those fish passed through the compromised gate at the outer end of the Little Bras d’Or Channel, while the third got through the gate on the northern end of the Barra Strait that connects the North and South basins of the Bras d’Or. These missed detections represent less than 9.5% of the total number of fish tag passages that were detected on these two gates (Table S1). It is important to note that this could be due to the fish rapidly swimming by the receivers and passing out of range before the tags transmitted, and may have little to do with actual detection efficiency.

Taken together, we are confident that our detection efficiency was highly satisfactory for achieving our research aims, and impose only a small level of uncertainty on our estimates of the various migration frequencies and patterns observed.

Table S1 | Detection efficiencies of Vemco VR2W acoustic receiver gates in the Bras d’Or Array of the Atlantic Ocean Tracking Network. The lowest efficiencies (expressed as the percentage of acoustic tag pings that were detected at each gate) are reported for the test tag type and conditions under which they occurred. Sentinel tags operated from 2011 to 2013, and range testing was undertaken during summer and autumn of 2011 and summer of 2012 using V9 tags. The numbers of acoustically tagged Atlantic salmon (*Salmo salar*) smolts that were known to have passed through gates without being detected are reported for the 2012 & 2013 tagged populations.

| **Receiver location** | **Position** | **N^o^ receivers** | **Sentinel tag**  **type** | **% of detections** | **Range test conditions (V9)** | **% of detections** | **Missed fish (V8) # (%)** |
| --- | --- | --- | --- | --- | --- | --- | --- |
|  |  |  |  |  |  |  |  |
| Nyanza Bay | mouth | 3 | V13 | 98.6 | surf, wind | 96.6 | 0 (0) |
| Little Narrows | west | 1 |  |  | surf, tide | 94.2 | 0 (0) |
|  | east | 1 |  |  | surf, tide | 90.9 | 0 (0) |
| Great BdO Channel | inner | 3 | V16 | 92.8 | bott, strat | 93.8 | 0 (0) |
|  | outer | 4 |  |  | surf, tide | 91.0 | 0 (0) |
| Deny’s Basin | west | 1 |  |  | surf, strat | 94.6 | n.d. |
|  | east | 1 |  |  | surf, strat | 96.2 | n.d. |
| Little BdO Channel | inner | 3 | V13 | 95.8 | bott, tide | 90.8 | 0 (0) |
|  | outer | 2 (1)* |  |  | bott, noise | 82.4 | 2 (9.5) |
| Barra Strait | north | 2 | V16 | 94.9 | surf, wind | 87.5 | 1 (4.3) |
|  | south | 2 |  |  | bott, wind | 89.7 | 0 (0) |
| St. Peter’s Channel | inner | 1 |  |  | bott, strat | 87.4 | n.d. |
|  | outer | 1 |  |  | surf, wind | 93.1 | n.d. |
|  |  |  |  |  |  |  |  |

See Fig. 1 for receiver locations. * One of the two receivers on the outer gate of the Little Bras d’Or Channel was lost during 2013. Conditions of lowest detection efficiency during range testing: surf = surface tag; bott = near seabed tag; strat = stratified water column; tide = Spring flow; wind = high wind-wave; noise = industrial & boat operations.
